# Malignancies among newly diagnosed systemic lupus erythematosus patients and their survival

**DOI:** 10.1177/09612033221131501

**Published:** 2022-10-06

**Authors:** Simo Kariniemi, Vappu Rantalaiho, Lauri J Virta, Hannu Kautiainen, Kari Puolakka, Pia Elfving

**Affiliations:** 1 School of Medicine, 101232 University of Eastern Finland, Kuopio, Finland; 2 60670 Jyväskylä Central Hospital, Jyväskylä, Finland; 3 8054 Kanta-Häme Central Hospital, Hämeenlinna, Finland; 4 Faculty of Medicine and Health Technology, 60650 Tampere University, Tampere, Finland; 5 Centre for Rheumatic Diseases, 162224 Tampere University Hospital, Tampere, Finland; 6 Research Department, Social Insurance Institution, Turku, Finland; 7 Primary Health Care Unit, 60650 Kuopio University Hospital, Kuopio, Finland; 8 Folkhälsan Research Center, Helsinki, Finland; 9 Terveystalo Healthcare, Lappeenranta, Finland; 10 Department of Medicine, 60650 Kuopio University Hospital, Kuopio, Finland; 11 Institute of Clinical Medicine, 101232 University of Eastern Finland, Kuopio; Finland

**Keywords:** systemic lupus erythematosus, malignancy, cancer, survival

## Abstract

The objective of this study was to evaluate the incidence of malignancies among newly diagnosed systemic lupus erythematosus (SLE) patients compared to reference individuals. Another aim was to assess the survival of SLE patients with malignancy compared to references with malignancy. Finnish adult (>17 years) newly diagnosed SLE patients were identified by their drug reimbursement decisions made during 1.1.2000–31.12.2014 from the register of the Social Insurance Institution. For each case, three population controls were individually selected by age, sex and place of residence. Overall, 1006 SLE patients (women 84%), with a mean age of 45.5 years (SD 16 years) and 3005 population controls were linked to Finnish Cancer Registry, and the information about incident malignancies was retrieved from the day the special reimbursement decision for SLE medication was accepted (index day, ID) until 31.12.2018 or until death. The patients diagnosed with malignancy were followed up until 31.12.2019 considering survival. During the follow-up, 85 SLE patients (women 78%) and 192 controls (women 78%) had developed one or more malignancy after the ID. The incidence rate ratio for any malignancy was 1.41 (95% CI 1.08–1.85). The most common malignancy in SLE patients was non-Hodgkin lymphoma, with twelve cases. SLE patients with malignancy had a lower adjusted 15-year survival than controls with malignancy, 27.1% versus 52.4%, and the adjusted hazard ratio for death was 1.68 (95% CI 1.17–2.43). Our results confirm that SLE patients have a higher risk for overall malignancy. The results also suggest that SLE patients with malignancy have lower survival than their references with malignancy.

## Introduction

Systemic lupus erythematosus (SLE) is a complex and chronic multi-organ autoimmune disease affecting primarily women.^1^ Systemic lupus erythematosus is also related to a great number of comorbidities, such as cardiovascular diseases, infections and mood disorders.^2,3^ Moreover, people with rheumatic diseases have a slightly higher risk for overall malignancy, and SLE is not an exception.^2,4–9^ Previous studies have shown that especially lymphomas and lung cancer are overrepresented among SLE patients.^2,4^

It has also been shown that SLE patients have a decreased overall survival due to lupus activity, comorbidities and some of the medications used compared to the general population.^10–12^ On the other hand, it seems that SLE patients do not experience higher mortality due to malignancy in general, but certain malignancies, such as haematological malignancies, may predispose SLE patients to higher mortality.^9–13^ However, it is likely that SLE and malignancy combined influence survival markedly, and SLE may be a risk factor for worse survival in the presence of malignancy.^14^ To our knowledge, this subject has seldom been studied.^14,15^

Thus, our aim was to depict the spectrum, number and risk of malignancies among incident SLE patients compared to reference individuals in Finland. We also aimed to assess the combined influence of SLE and malignancy on survival in this large register-based study.

## Methods

Every permanent inhabitant in Finland has National Health Insurance, and the Finnish Social Insurance Institution (SII) holds a register of these insurances. SLE patients were retrieved for this study based on new reimbursement decisions of SLE medication costs during 1.1.2000–31.12.2014. The patients were identified by the World Health Organization’s (WHO) 10th International Classification of Diseases (ICD-10) code of M32. The date of acceptance of reimbursement was defined as the date of SLE diagnosis (index date, ID), and it was the same for the controls.

We performed a nationwide case-control study consisting of only adults (age >17 years). For every incident SLE patient, three individually matched population controls (age, sex and place of residence at the ID) were randomly selected from the Population Register Centre. The education level was determined at baseline (basic, middle, lower high and upper high level) from information acquired from Statistics Finland.

Every new malignancy has been reported to the Finnish Cancer Registry starting from the year 1953. Besides definite malignancies, the registry includes in situ – cancers, high-grade squamous intraepithelial lesions (HSIL) and severe dysplastic alterations (except for skin cancers, where only melanoma in situ alterations are reported), ovarian tumours graded as borderline change and benign central nervous system (CNS) tumours. Moreover, some other disease states, the malignancy of which is considered unclear (such as polycythaemia vera, myelofibrosis and neuroendocrinal tumours) are recorded. Also, tumours that are highly suspected as malignant, even though no microscopic confirmation is at hand, are reported. The malignancies are reported according to the WHO`s ICD-10 codes or according to International Classification of Diseases for Oncology codes (ICD-O-3). No relapses have been recorded for this registry.^16^

The information regarding the incident malignancies was retrieved between 1.1.2000 and 31.12.2018 with the follow-up starting from the ID of each patient and lasting until 31.12.2018 or until the patient died, whichever occurred first. Malignancies that were diagnosed before the ID were excluded from this study. The survival of patients with malignancy was followed up until 31.12.2019, and it was adjusted by age, sex and education.

The reported malignancies were classified in 13 groups according to the literature as follows: breast cancer, prostate cancer, lung cancer, cancers of colon and rectum, melanoma, non-melanoma skin cancer (NMSC), haematologic malignancy (consisting of leukaemias, myelofibrosis, myeloma and polycythaemia vera), bladder cancer, stomach cancer, pancreatic cancer, non-Hodgkin lymphoma (NHL), gynaecological cancer (including cancers of cervix and corpus uteri and vulva) and other cancers (including cancers of CNS, nerve sheet and eye, meningiomas, kidney cancers, Hodgkin lymphoma, other gastrointestinal-tract cancers and gallbladder, biliar duct and hepatic cancers, cancers of salivary and thyroid glands, mesotheliomas, cancers of testis and upper respiratory tract). In addition, malignancies that were ill-defined or unknown were classified into the ‘other’ group.

In Finland, causes of death of all permanent Finnish residents are recorded to the causes of death statistics maintained by Statistics Finland. The causes of death are registered in four groups as follows: underlying cause of death, immediate cause of death, intermediate cause of death and contributory causes of death. The underlying cause of death is the disease that initiates the course leading to death, and it is used in official annual death certificate registers. The causes of death are recorded according to ICD-10 codes on the death certificate, and the certificate is written by the physician, who has been the last to treat the deceased patient. Every certificate is checked by a forensic pathologist from the Finnish Institute of Health and Welfare afterwards.^17^

Our aim was to evaluate the number of malignancies (C00-D48) as underlying causes of death among SLE patients and references. Furthermore, as causes of death, we inspected eight other ICD-10 groups of special interest as follows: certain infectious and parasitic diseases (A00-B99), mental and behavioural disorders (F00-F99), diseases of the nervous system (G00-G99), diseases of the circulatory system (I00-I99), diseases of the respiratory system (J00-J99), diseases of the digestive system (K00-K93) and symptoms, signs and abnormal clinical and laboratory findings, not elsewhere classified (R00-R99).

Since this study was register-based and done without contacting study subjects, neither approval of an ethical committee nor the patient`s informed consent was required by Finnish law.

## Statistical methods

The characteristics were presented as means with standard deviation (SD) for continuous variables and as frequencies with percentages for categorical variables. The incidence of malignancy rates (per 1000 person years) with 95% confidence intervals (CIs) were calculated assuming Poisson distribution; number of events with person-years. Incidence rate ratios (IRRs) were calculated using Poisson regression models, or negative binomial regression models when appropriate. The assumptions of overdispersion in Poisson model were tested using the Lagrange multiplier test. The Kaplan–Meier method was used to estimate the cumulative incidence and log-rank test to assess differences between groups. The adjusted Kaplan–Meier cumulative survivals were estimated using inverse probability of treatment weighting.^18^ Cox proportional hazards regression was used to estimate the hazard ratios (HRs) and their 95% CIs. The proportional hazards assumption was tested graphically and by use of a statistical test based on the distribution of Schoenfeld residuals. All statistical analyses were carried out with Stata version 17.0 (StataCorp, College Station, TX).

## Results

The study included 1006 SLE patients (mean age 45.5, SD 16 years, females 84%) and 3005 controls. Mean ages were 44.9 (SD 15.9) years in women and 48.6 (SD 16.4) years in men. Among SLE patients, follow-up was a total of 11,294 person-years, 1512 in men and 9782 in women, resulting in a mean of 11.2 years of follow-up for any malignancy. Similarly, among controls, follow-up was a total of 34,734 person-years, 4875 in men and 29,858 in women.

During the follow-up, 85 patients with SLE (78% women) developed a malignancy, whereas in controls the number was 192 (78% women). Seven SLE patients (five women and two men) and 15 controls (11 women and four men) developed more than one malignancy during the follow-up. Compared to controls, SLE patients had a significantly higher IRR for overall malignancy among all patients and women, with IRRs 1.41 and 1.40, respectively (Tables 1 and 2). However, men with SLE did not differ significantly from the controls, likely due to the small number of cases (Table 3).

**Table 1.** Numbers, incidence rates per one thousand person-years and incidence rate ratios of recorded malignancies in newly diagnosed systemic lupus erythematosus patients and controls in Finland from the index day until 31.12.2018 or until the patient died, whichever occurred first. Sex-specific malignancies are presented in Table 2 (women) and in Table 3 (men).

		SLE patients		Controls	
Malignancy	N	IR per 1000 95% CI	N	IR per 1000 95% CI	IRR 95% CI
Any malignancy	96	8.5 (6.6–10.4)	209	6.0 (5.2–6.9)	1.41 (1.08–1.85)
Lung	8	0.7 (0.2–1.2)	11	0.3 (0.1–0.5)	2.24 (0.90–5.55)
Colorectal	8	0.7 (0.2–1.3)	12	0.3 (0.2–0.5)	2.05 (0.79–5.34)
Melanoma	0	0.0	13	0.4 (0.2–0.6)	
NMSC	8	0.7 (0.0–1.5)	7	0.2 (0.1–0.4)	3.51 (0.94–13.16)
Haematologic	10	0.9 (0.3–1.4)	15	0.4 (0.2–0.6)	2.05 (0.92–4.56)
Bladder	1	0.1(0.0–0.3)	16	0.5 (0.2–0.7)	0.19 (0.03–1.45)
Stomach	0	0.0	9	0.3 (0.1–0.4)	
Pancreas	5	0.4 (0.1–0.8)	4	0.1 (0.0–0.2)	3.84 (1.03–14.31)
Non-Hodgkin lymphoma	12	1.1 (0.5–1.7)	7	0.2 (0.1–0.4)	5.27 (2.08–13.36)
Other	21	1.9 (1.0–2.7)	34	1.0 (0.7–1.3)	1.90 (1.09–3.31)

SLE = systemic lupus erythematosus; N = number; IR: incidence rate; IRR = incidence rate ratio.

Note: Lung = lung cancer; colorectal = cancers of colon and rectum; melanoma; NMSC = non-melanoma skin cancer; haematologic = haematologic malignancy consisting leukaemias, myelofibrosis, myeloma and polycythaemia vera; bladder = bladder cancer; stomach = stomach cancer; pancreatic = pancreatic cancer; Non-Hodgkin lymphoma; other = other cancers including cancers of CNS, nerve sheet and eye, meningiomas, kidney cancers, other GI-tract cancers and gallbladder, biliar duct and hepatic cancers, cancers of salivary and thyroid glands, mesotheliomas, cancers of testis and upper respiratory tract and cancers that were ill-defined or unknown.

**Table 2.** Numbers, incidence rates per one thousand person-years and incidence rate ratios of recorded malignancies in women with newly diagnosed systemic lupus erythematosus and control women in Finland from the index day until 31.12.2018 or until the patient died, whichever occurred first.

		SLE patients		Controls	
Malignancy	N	IR per 1000 95% CI	N	IR per 1000 95% CI	IRR (95% CI)
Any malignancy	75	7.7 (5.7–9.7)	163	5.5 (4.6–6.3)	1.40 (1.03–1.91)
Breast	11	1.1 (0.5–1.8)	60	2.0 (1.5–2.5)	0.56 (0.30–1.06)
Lung	6	0.6 (0.1–1.1)	8	0.3 (0.1–0.5)	2.29 (0.80–6.58)
Colorectal	7	0.7 (0.1–1.3)	9	0.3 (0.1–0.5)	2.37 (0.82–6.89)
Melanoma	0	0.0	12	0.4 (0.2–0.6)	
NMSC	8	0.8 (0.0–1.7)	4	0.1 (0.0–0.3)	6.10 (1.40–26.49)
Haematologic	7	0.7 (0.2–1.2)	8	0.3 (0.1–0.5)	2.67 (0.97–7.35)
Bladder	1	0.1 (0.0–0.3)	11	0.4 (0.2–0.6)	0.28 (0.04–2.15)
Stomach	0	0.0	5	0.2 (0.0–0.3)	
Pancreas	3	0.3 (0.0–0.7)	4	0.1 (0.0–0.3)	2.29 (0.51–10.22)
Non-Hodgkin lymphoma	9	0.9 (0.3–1.5)	4	0,1 (0.0–0.3)	6.87 (2.12–22.25)
Gynaecological	7	0.7 (0.2–1.2)	11	0.4 (0.2–0.6)	1.94 (0.76–5.00)
Other	16	1.6 (0.8–2.5)	27	0.9 (0.6–1.2)	1.81 (0.96–3.42)

SLE = systemic lupus erythematosus; N = number; IR: incidence rate; IRR = incidence rate ratio.

Note: Breast = breast cancer; lung=lung cancer; colorectal = cancers of colon and rectum; melanoma; NMSC = non-melanoma skin cancer; haematologic = haematologic malignancy consisting leukaemias, myelofibrosis, myeloma and polycythaemia vera; bladder = bladder cancer; stomach = stomach cancer; pancreatic = pancreatic cancer; Non-Hodgkin lymphoma; gynaecological = gynaecological cancer including cancers of cervix and corpus uteri and vulva; other = other cancers including cancers of CNS, nerve sheet and eye, meningiomas, kidney cancers, other GI-tract cancers and gallbladder, biliar duct and hepatic cancers, cancers of salivary and thyroid glands, mesotheliomas, cancers of testis and upper respiratory tract and cancers that were ill-defined or unknown.

**Table 3.** Numbers, incidence rates per one thousand person-years and incidence rate ratios of recorded malignancies in men with newly diagnosed systemic lupus erythematosus and control men in Finland from the index day until 31.12.2018 or until the patient died, whichever occurred first.

		SLE patients		Controls	
Malignancy	N	IR per 1000 95% CI	N	IR per 1000 95% CI	IRR (95% CI)
Any malignancy	21	13.9 (7.7–20.1)	46	9.4 (6.6–12.3)	1.47 (0.86–2.52)
Breast	0	0.0 (0,0 to 0,0)	0	0.0 (0.0–0.0)	
Prostate	5	3.3 (0.5–6.1)	10	2.1 (0.8–3.3)	1.61 (0.56–4.61)
Lung	2	1.3 (0.0–3.2)	3	0.6 (0.0–1.3)	2.15 (0.36–12.89)
Colorectal	1	0.7 (0.0–2.0)	3	0.6 (0.0–1.3)	1.07 (0.11–10.27)
Melanoma	0	0.0	1	0.2 (0.0–0.6)	
NMSC	0	0.0	3	0.6 (0.0–1.3)	
Haematologic	3	2.0 (0.0–4.2)	7	1.4 (0.4–2.5)	1.38 (0.36–5.32)
Bladder	0	0.0	5	1.0 (0.1–1.9)	
Stomach	0	0.0	4	0.8 (0.0–1.6)	
Pancreas	2	1.3 (0.0–3.1)	0	0.0	
Non-Hodgkin lymphoma	3	2.0 (0.0–4.2)	3	0.6 (0.0–1.3)	3.22 (0.65–15.90)
Other	5	3.3 (0.4–6.2)	7	1.4 (0.4–2.5)	2.30 (0.74–7.18)

SLE = systemic lupus erythematosus; N = number; IR = incidence rate; IRR = incidence rate ratio.

Note: Breast = breast cancer; prostate = prostate cancer; lung = lung cancer; colorectal = cancers of colon and rectum; melanoma; NMSC = non-melanoma skin cancer; haematologic = haematologic malignancy consisting leukaemias, myelofibrosis, myeloma and polycythaemia vera; bladder = bladder cancer; stomach = stomach cancer; pancreatic = pancreatic cancer; Non-Hodgkin lymphoma; other = other cancers including cancers of CNS, nerve sheet and eye, meningiomas, kidney cancers, other GI-tract cancers and gallbladder, biliar duct and hepatic cancers, cancers of salivary and thyroid glands, mesotheliomas, cancers of testis and upper respiratory tract and cancers that were ill-defined or unknown.

A significantly increased risk for NHL, pancreatic cancer and other malignancies was recorded (Table 1). The most common malignancy in SLE patients was NHL, with twelve cases. For the other haematologic malignancies, the spectrum varied widely between different types of leukaemia, myeloma and other types of malignant blood diseases. Moreover, NMSC, colorectal and lung cancers were prevalent in SLE patients. Interestingly, no melanomas were recorded among SLE patients compared to 13 melanomas in control patients.

Breast cancer was common among both SLE patients and controls in women, but no statistical difference was recorded between the groups (Table 2). Instead, women with SLE had significantly increased risk for NHL and NMSC. In men with SLE, prostate cancer was the most common malignancy, but no significant difference was recorded for any malignancy compared to controls (Table 3).

**Figure 1.**
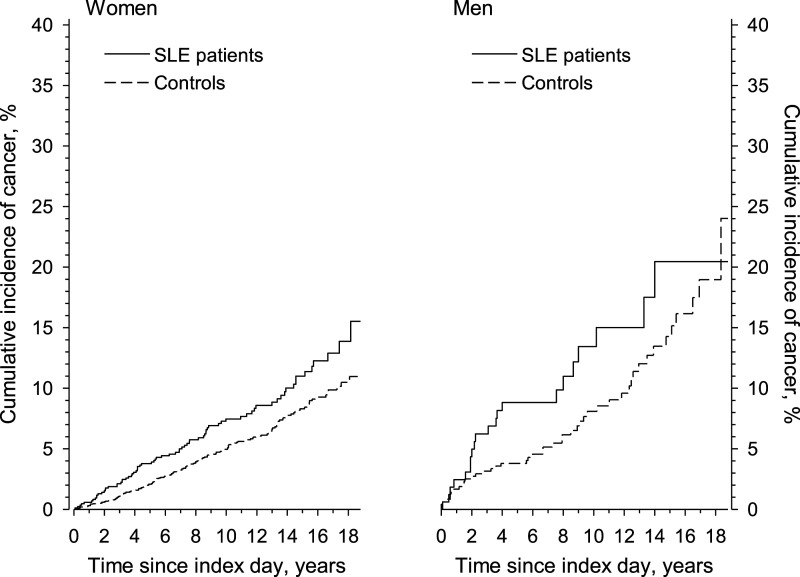
Cumulative incidence of malignancies along time in newly diagnosed systemic lupus erythematosus patients and controls by sex in Finland from the index day until 31.12.2018 or until the patient died, whichever occurred first.

Malignant cases appeared steadily among women with SLE through the follow-up. Moreover, the cumulative incidence of malignancy among women with SLE started to differ 1 year after the ID, and the relative difference persisted over time compared to control women (Figure 1).

**Figure 2.**
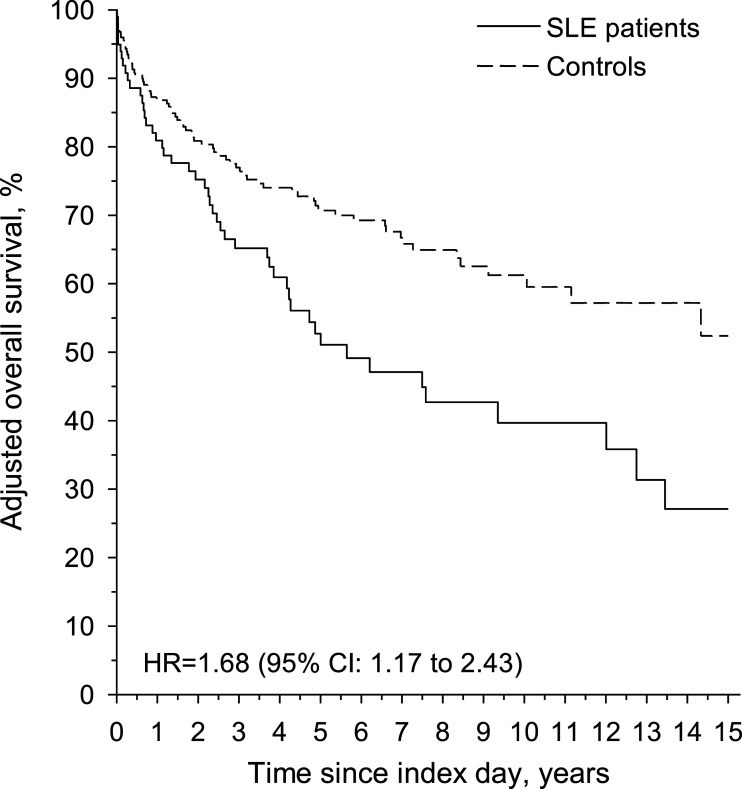
Adjusted (age, sex and education) overall survival in newly diagnosed systemic lupus erythematosus patients and controls with a malignancy from the index day until 31.12.2019.

Altogether, 122 of the 277 persons who developed a malignancy during the follow-up died. Deaths were more frequent among SLE patients (N = 48) than among controls (N = 74). By the end of the follow-up, the crude survival for persons with malignancy was 30.0% (95% CI 17.4%–43.6%) in SLE patients and 47.2% (95% CI 33.9%–59.4%) in controls, *p* = 0.020. The age-, sex- and education-adjusted 15-year survival was 27.1% and 52.4% for the SLE patients and controls, respectively (Figure 2), and the adjusted HR for death was 1.68 (95% CI 1.17–2.43).

The most common cause of death among patients with malignancy was malignancy among both SLE patients (N = 34, 70%) and controls (N = 56, 76%). Nine patients (19%) and seven controls (9%) died of cardiovascular diseases. The rest of the causes of death were divided evenly (data not shown). Infection was marked as a contributory cause of death in four patients (8%) and two controls (3%), and SLE in six patients (13%).

## Discussion

In this large nationwide case-control study, we found the incidence of overall malignancies to be slightly higher among newly diagnosed SLE patients than among population controls in Finland. Although the rates for specific malignancies were quite low, we found a significantly increased risk for NHL. The risk of NMSC was also higher among women with SLE, but interestingly no cases of melanoma were found in SLE patients. SLE patients with any malignancy also had a distinctively worse survival than references with any malignancy.

We found that the risk of developing a malignancy was almost 1.5-fold higher among SLE patients. This finding is in line with previous studies, which have reported standardised mortality ratios for developing any malignancy ranging from 1.1 to 1.9 in SLE.^4,19,20^ Moreover, in a nationwide Korean study from 2008 to 2014, an odds ratio of 1.4 for any cancer was recorded in newly diagnosed SLE.^21^

In our study, the most common malignancy was NHL in SLE patients. In addition, the second most common malignancy was breast cancer, but we did not record any significant difference in breast cancer between SLE patients and controls. Altogether, our study results do not differ from other studies by much. Especially NHL and lung cancer have been overrepresented among SLE patients, while no increased risk has been recorded for some other types of malignancies, such as breast cancer.^4,9,19^ Moreover, in another Finnish study with almost 26 years of follow-up and conducted between 1967 and 2013, Tallbacka et al. found the risk of overall malignancy in SLE to be nearly doubled. They also found an increased risk for NHL and kidney cancer. However, they did not find an elevated risk for NMSC or pancreatic cancer, as we did. The minor differences between our study results and theirs may be explained by the longer follow-up time, smaller sample size and the fact that they included only SLE patients treated at Helsinki University Central Hospital.^20^

The most notable difference between SLE patients and their references was recorded for NHL risk as it was more than five times higher in SLE patients in our study. It has been shown that some of the autoimmune diseases are related to certain types of haematologic malignancies possibly due to deficiencies in immunoregulation.^9,22,23^ SLE patients seem to be particularly prone to develop a lymphoma.^4,9,22–28^ For instance, Bernatsky et al. showed three and four times higher risks for all haematologic malignancies and for all lymphomas, respectively, in their large international multicentre (USA, Canada, Europe and South Korea) cohort study.^4^ Reasons for increased lymphoma risk are unknown, but chronic inflammation, chromosomal abnormalities, different kinds of cytokines, immunosuppressive treatment and disease activity may have a role in the pathogenesis.^4,9,22–25^ In particular, SLE patients seem to be prone to NHL, as an over four times higher risk has been depicted.^4,26–28^

Interestingly, we found an elevated risk for NMSC among women with SLE, but no melanomas were recorded among SLE patients. Our results are similar to other studies from the Nordic countries which have shown the NMSC risk to be slightly increased in SLE.^29,30^ It has been proposed that the increased risk could partly result from the use of cyclophosphamide. In contrast, the use of hydroxychloroquine could be a protective factor.^23,31^ A slightly decreased risk for melanoma in SLE patients has been reported in a study from the state of California, whereas Bernatsky et al. showed no significant difference in risk.^4,32^ We presume that the increased NMSC risk may partly be explained by immunosuppressive medication in our study, although we lacked the clinical information. On the other hand, the reduced melanoma risk could be explained by the decreased sun exposure among SLE patients to some extent.^33^ However, a surveillance bias considering both NMSC and melanoma is possible, since SLE patients and their skin are likely followed up more closely than other people.

We found that malignancy in the lungs was one of the most frequent single malignancy types, but no significant difference was recorded compared to controls among all patients or observed along sex. Our study result differed from others who have found the lung cancer risk to be approximately twice as high in SLE.^4,19,29,32^

A significantly increased risk for pancreatic cancer was found in our study, but the number of cases was limited. Previous reports on the risk of pancreatic cancer in SLE are not uniform^4,19–21,26,32^, and further studies are needed to determine the actual risk.

We found only sporadic cases of gynaecological cancers of varying origin, as did Tallbacka et al.,^19^ and we could not confirm any significant increased risk. Earlier findings on gynaecological cancers are not consistent.^4,20,21,32,34^ Some studies have shown a higher risk for vulvar or vaginal cancer, whereas cancer risk of uterus and ovaries seem to be reduced.^4,21,32,34^ Moreover, women with SLE may have an increased risk for cervical neoplasia, but the risk of developing cervical cancer is not well established.^4,21,33–35^ Our study result may be partly explained by the extensive cervical cancer screening program in Finland, which prevents progression to cancer in some cases.^9,23,36,37^

Our other aim was to compare the survival of SLE patients with malignancy to general population controls with malignancy and to assess SLE as a risk factor for worse survival in coincidence with any malignant disease. We found that malignancy was the most common cause of death among both SLE patients and controls with malignancy. In our study, the risk of death was almost two-fold higher among SLE patients with a malignant disease, suggesting that SLE impairs survival among people with a malignancy. We pondered that the decreased survival may partly be explained by the complex immune dysregulation and the medication used for SLE, which both predispose to infections.^38–40^

Our study result is in line with a large study from the United States, which compared the survival of elderly women with both SLE and breast cancer to women with breast cancer or SLE alone. They discovered that patients with both SLE and cancer had a higher mortality than patients with cancer or SLE alone.^14^ Moreover, one retrospective cohort study evaluated the survival of patients with both rheumatic disease and cancer compared to the general population. They showed that in certain rheumatic diseases (dermatomyositis, polymyositis and rheumatoid arthritis), the survival was decreased in coincidence with cancer. However, the number of SLE patients was limited in the study.^15^

The strengths of this study are the case-control study design and the mean follow-up of more than 11 years. The data on the incidence of malignancy and causes of death were retrieved from official registers, the reliability of which is well established and regularly monitored.^41^ We also linked many different official registers and used extensive nationwide data, including all incident malignancies diagnosed both in primary and specialised care. We included all newly diagnosed SLE patients during 1.1.2000–31.12.2014 in Finland.

A major limitation of this study is the lack of clinical data. Therefore, we could not determine the severity of SLE. In addition, we were not able to investigate the effect of many acknowledged confounding factors such as smoking habits and obesity on the risk of malignancy. We also lacked the specific diagnoses and details of some malignancies, such as the stage of malignancy. There may also be some surveillance bias because SLE patients are regularly monitored.

In conclusion, we showed that SLE patients had a higher risk for overall malignancy. Especially the risk of NHL was elevated. We also showed that SLE patients with any malignancy had a worse survival than the references with malignancy. Our study results demonstrated an increased risk for certain, sometimes unscreenable, malignancies that emphasises the importance of early clinical suspicion and diagnosis.
